# Physical and chemical properties of dust in the Pre-Aral region of Uzbekistan

**DOI:** 10.1007/s11356-022-18827-6

**Published:** 2022-01-27

**Authors:** Rustam Bazarbayev, Biao Zhou, Atabek Allaniyazov, Guanggen Zeng, Damir Mamedov, Evgenia Ivanitskaya, Qingzhu Wei, Hongqiang Qian, Komiljon Yakubov, Mohsen Ghali, Smagul Karazhanov

**Affiliations:** 1 Department of Physics and Mathematics, Urgench State University, Urgench, Uzbekistan; 2 College of Materials Science and Engineering, Sichuan University, Chengdu, 610065 China; 3 Department of Physics, Karakalpak State University, Nukus, Republic of Karakalpakstan Uzbekistan; 4 Department for Solar Energy, Institute for Energy Technology, NO-2027 Kjeller, Norway; 5 Department of Materials Science, National Research Nuclear University (MEPhI), Moscow, Russia; 6 Suzhou Talesun Solar Technologies Co., Ltd, Suzhou, 215542 Changshu China; 7 School of Basic and Applied Sciences, Egypt-Japan University of Science and Technology, Alexandria, Egypt

**Keywords:** Desert dust, Aralkum Desert, Aral Sea, Pre-Aral region, Composition and size of dust particles, Influence of dust on human, Soiling of photovoltaic modules, Physical and chemical properties of soiling

## Abstract

The aim of this work is study of physical and chemical properties of dust of the Pre-Aral region of Uzbekistan such as Karakalpakstan and Khorezm that are located near the three deserts such as the Aralkum, Karakum, and Kyzylkum. The dust particles fell on glass have been collected in Karakalpakstan and Khorezm and studied systematically by employing wide range of methods. Particle volume vs size distribution has been measured with maximum around 600 nm and ~ 10 µm. The major and minor constituent materials present in the dust have been studied systematically by X-ray fluorescence spectroscopy, energy dispersive X-ray diffraction, and inductively coupled plasma optical emission spectroscopy. Main characteristic absorption bands corresponding to Si–O, Si–O-Si bonding in quartz and Fe–O bonds in hematite Fe_2_O_3_ have been identified by infrared and Raman spectroscopy. Quartz, hematite, lime, corundum, magnesia, and several other trace minerals have been identified in the dust particles. X-ray diffraction peaks corresponding to quartz, hematite, and corundum are sharp and are found to be more crystalline with some level of disorder. Analysis of the particle size and crystallinity on human being has been performed: disordered or crystalline quartz can create the lung disease; the particles in the size of 0.5–0.7 µm may produce diseases such as chronic silicosis, silicosis, and silica tuberculosis whereas hematite might create lung disease. Dust particles worsen optical transmittance of glass of the panels.

**Graphical abstract:**

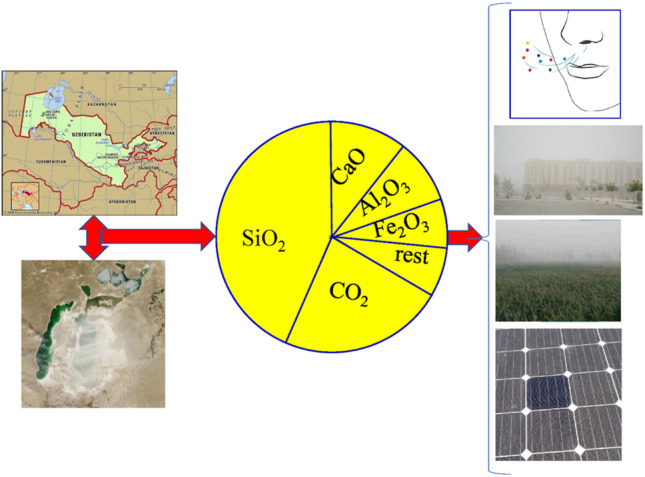

## Introduction

Because of climate change and industry-related activity, air pollution is becoming a major concern (Li et al. 2009, Tumwesige et al. 2017, WHO 2020). The inhaled air pollutants may cause series health problems and lead to diseases, such as high blood pressure, nerve and kidney disorders, pulmonary and heart diseases, memory and concentration problems, and muscle and joint pain, and it also affects the immune systems and may even lead to cancer (Senthil Kumar and Rajkumar 2013).

The Pre-Aral regions of Uzbekistan such as Karakalpakstan and Khorezm are surrounded by three deserts. One of them is the newly formed Aralkum, formed because of drying out the former Aral Sea that caused severe climate change. The other two deserts are Kyzyl Kum Desert located in the east side and the Karakum Desert in the south. Previously, the Aral Sea was blocking the wind from the North. After drying out of the large parts of the Sea, a salty desert—the Aralkum—has been formed that is subjected to wind erosion and results in white sand and dust storms. The problem of air pollution and climate change strongly influences human health, environment, and technical infrastructure, in particular, solar panel installations (Avezova et al. 2019, Groll et al. 2019). Now, the pre-Aral Sea region is an extreme continental climate area. The temperature in this region can exceed 45 °C in summer. The salts from the Aralkum are coming by sandstorm every year and the ecology is fragile. High frequency and intensity dust emissions are reported (Nobakht et al. 2019) in the Pre-Aral region from the Aralkum, Karakum, and Kyzylkum Deserts. So, occurrence of respiratory diseases in this area resulting from air pollution can be quite substantial. Thus, it is crucial to study physical and chemical properties of dust, identifying components of the dust, and eliminating the pollutants that negatively influence human. The air pollution induced by climate change in the Pre-Aral region influences not only human being and environment, but also technical infrastructure. The increasing demand in energy resources has led to widespread solar panel installations in many geographical locations. One of the main challenges faced is the high operating temperature during summer days and the soiling from air pollution that worsens performance of solar modules (Bergin et al. 2017, Boyle et al. 2015, Costa et al. 2016, Lopez-Garcia et al. 2016, Maghami et al. 2016, Sarver et al. 2013, Urrejola et al. 2016).

The studies related to influence of local climate conditions such as solar irradiation, wind and soiling speed, dust storm, temperature, air pollution, and humidity on degradation of solar panels always were under the focus of intensive research in the last more than two decades (Alquthami and Menoufi 2019, Chanchangi et al. 2021, Cordero et al. 2018, Costa et al. 2016, Maghami et al. 2016, Sarver et al. 2013, Urrejola et al. 2016). Many authors have addressed (Boyle et al. 2015, Javed et al. 2017) the study of physical nature of dust and attempted to establish connection between the dust parameters, deposition of the dust, and solar panel performance. Study of soiling in different geographical locations has been the subject of numerous studies for Atacama Desert in South America (Cordero et al. 2018); Qatar (Aïssa et al. 2016); Santiago; Chile (Urrejola et al. 2016); Egypt (Alquthami and Menoufi 2019); and for Navarre, Spain (García et al. 2011). The aim of this paper is investigation of physical and chemical properties of dust particles in Nukus; Karakalpakstan; and Urgench, Khorezm, and Uzbekistan—the Pre-Aral regions that are subjected to strong influence of the abovementioned three Deserts. The study is important as the total sunshine hours in the Pre-Aral regions are close to 3000 h throughout the year, which has obvious advantages in photovoltaic power generation. Here, the knowledge of the dust elemental composition and particle size distribution might provide valuable information about developing the effective prevention techniques against pollution of solar panels by selecting the methods of cleaning of solar panels and proper anti-soiling coatings for the glass. It can also be important to eliminate the dust particles that are detrimental for human being.

## Experimental section

### Collection of dust particles

**Fig. 1:**
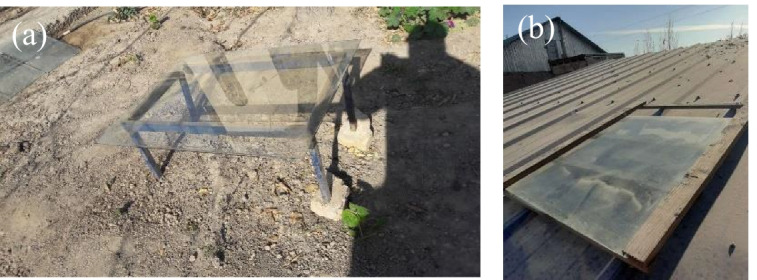
Setups used for dust collection in (**a**) 42°26′23.0"N 59°35′36.3"E, Nukus, Karakalpakstan and (**b**) 41°55′11.4"N 60°16′36.2"E, Gurlan, Khorezm, Uzbekistan

Glass plates commonly used in solar modules were placed outside in two cities of Uzbekistan. One of them is the city Nukus, Republic of Karakalpakstan. The other one was placed in the village Dosumbiy, Gurlan district, Khorezm, Uzbekistan located in ~ 170 km distance from Nukus. The glass sheet in Nukus city was placed (Fig. 1(a)) on 1-m distance from the Earth surface in a ~ 600-m^2^ private territory surrounded with walls. Solar panels are commonly installed in such private territories. The other one in Urgench was placed on roof of a building in rural region, ~ 2.5 m from the Earth surface (Fig. 1(b)). The dust particles were collected from the glass sheets every week carefully by brush and were stored in glass bottles. The glass sheets were reused for collecting the dust. No washing was applied. When it was snowing or raining, the dust was collected after it was dried out in natural conditions.

The dust particles collected in Khorezm and Karakalpakstan within 1 year have been used for compositional analysis. Weight of the dust was measured by electronic weight. A 2.5 × 7.5-cm^2^ microscopic glass was used to measure transmission spectra before and after dust deposition. Although the distance between Karakalpakstan and Khorezm is close, some difference was expected in physical and chemical properties of the dust particles collected in these two regions, but the difference was within the measurement accuracy.

### Characterization of dust particles

Dynamic light scattering (DLS) was performed using a Mastersizer for large size (accuracy < 1%) and Zetasizer nano ZS (Malvern Instruments Ltd., Worcestershire, UK) for smaller size (0.3 nm–10 µm) dust particles, respectively, to measure the size distribution of the dust nanoparticles. One can get intensity, volume, or number-based distributions from the instruments. The intensity-weighted distribution shows how well differently sized particles are detected from a fit to the autocorrelation function of the measured scattering and can therefore be highly sensitive to very small numbers of aggregates or dust, as scattering intensity is proportional to the 6th power of the particle radius. In this paper, we focus on the number-based distributions that show the relative proportion of number of differently sized particles by differently sized particles, which is enough for our purpose.

Water is used for dust dispersion. HCl was used to reduce pH and NaOH was used for increasing the pH. Size measurement and morphological investigation of the dust nanoparticles were characterized by scanning electron microscopy (SEM) JSM-5610LVS; JEOL, Tokyo, Japan. Elemental composition of the dust has been performed by energy dispersive X-ray diffraction (EDX). The samples were not pre-treated before the SEM and EDX measurements. Compositional analysis has also been performed by XRF (XRF-1800 produced by Shimadzu, Japan) that utilizes X-rays to determine the elemental composition of rocks, minerals, cement, ceramics, metals, and petroleum. The analysis determines the types of elements in the sample based on the characteristic wavelengths of the X-rays emitted by the atoms. Inductively coupled plasma optical emission spectroscopy (ICP-OES) Agilent 5100 SVDV ICP-OES is used for the detection of chemical elements. The optical characterization of the clean and dusty glass was investigated using a Perkin Elmer Lambda 35 UV/Vis Spectrophotometer, equipped with tungsten and deuterium light sources. The transmission measurements were calibrated with respect to 100% transmission in air. Powder X-ray diffraction (XRD) data were obtained using a Shimazu XRD-6100 diffractometer with Cu-Kα radiation (λ = 1.5406 Å) at 40 kV and 30 mA. Infrared (IR) spectra have been measured in transmission mode by Bruker INVENIO Germany with resolution of 4 cm^−1^ and scan times of 16 s. Raman produced by Horiba, France, has been used for the study of Raman spectra of the dust soiling accumulated on glass. High power 532 nm laser has been used for the Raman excitation, which is a workhorse for inorganic materials.

## Results and discussion

### Dust accumulation

**Fig. 2:**
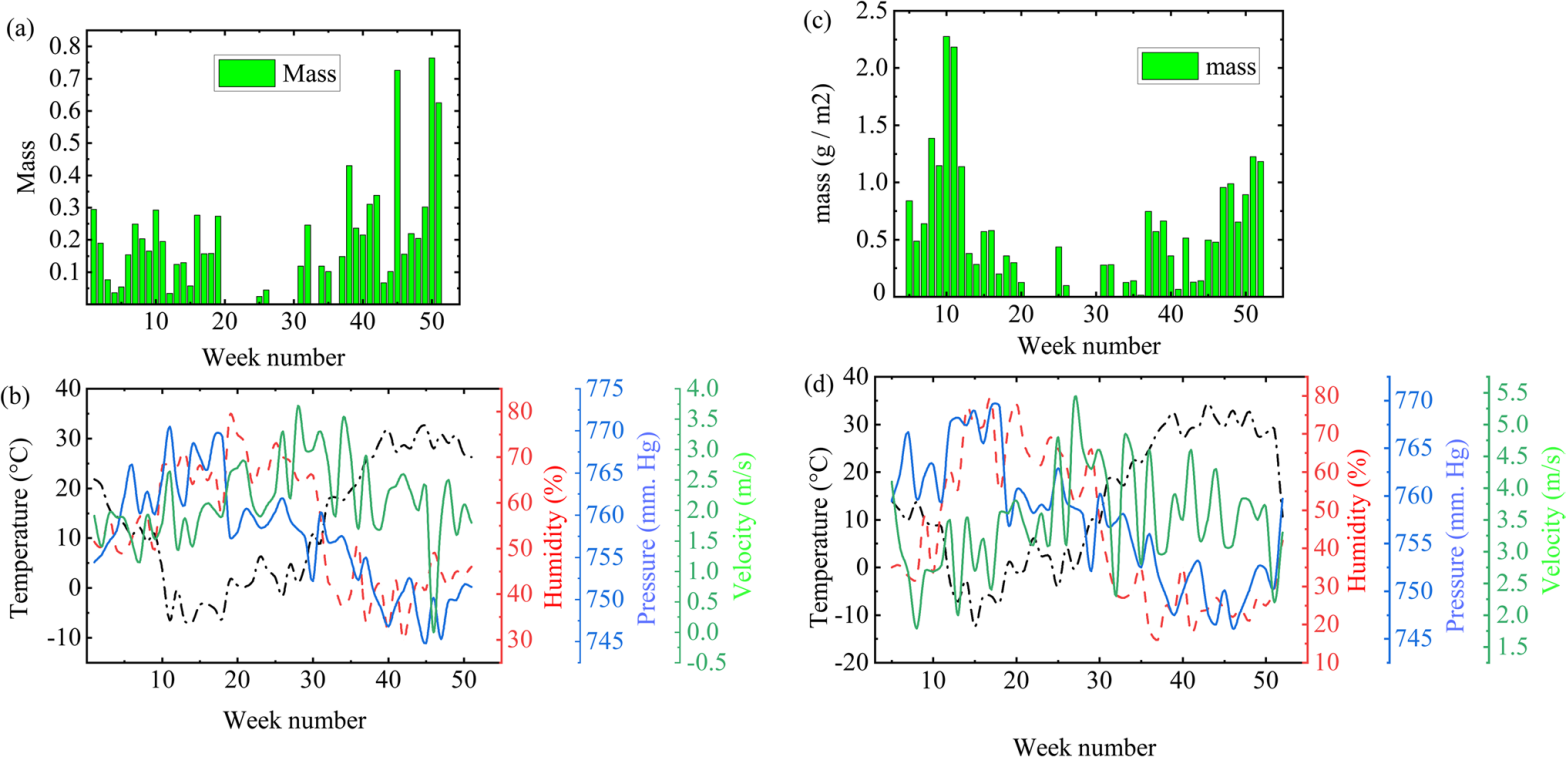
Mass of dust collected from the top of a 0.5 × 0.5-m^2^ area glass sheet placed in (**a**) Khorezm on roof of a building in rural region and (**c**) Karakakalpakstan in the city. The dust has been collected once per week from 09 January 2020. The number of weeks has been shown on abscess axes. **b** and **d** show the temperature, humidity, air pressure, and wind velocity in each local region

Figure 2 shows the dust accumulation in Urgench; Khorezm; and Nukus, Karakalpakstan. Analysis shows that the dust was accumulated with the rate between 160 and 1200 mg per m^2^ per week in Urgench in the village in open area and 80–1000 mg/m^2^ per week in Karakalpakstan in a private house in the city. The dust amount is enough to reduce transmittance of glass and performance of PV modules to a few percent (Sarver et al. 2013) within 1 day, which is critical. This indicates the importance of cleaning of PV modules twice per week at least. The observed dust accumulation rate is larger than 70–560 and 35–140 mg/(m^2^ week^−1^) in at Commerce City and the Erie site of CO, USA (Boyle et al. 2016), and is smaller than 1050–2100 mg/(m^2^ week^−1^) in the hot desert region Minya in Egypt (Hegazy 2001). The difference in dust accumulation rates in different regions can be explained (Cordero et al. 2018, Javed et al. 2017) by dependence of the dust accumulation rate on local environment, angle of deployment, time of year, wind speed, air temperature and pressure, and humidity. This complicates to find well-defined correlation between the dust mass per square meter on one of the above points and the dust accumulation rate within the year cannot be universal for all locations.

The dust accumulation rates have been plotted for 1 year from August, 2020 to August, 2021 (Fig. 2) for Urgench and Karakalpakstan—the Pre-Aral regions of Uzbekistan. Outside the human populated area, larger soiling rates are expected. Because of the climate range in the Pre-Aral region, the climate is often windy leading to well mixing and higher density of the air pollutants. Majority of the human height is within 1–2.5 m, so the dust pollutants can be well inhaled by human.

### Dust particle size, morphology, and pH in dispersion with water

**Fig. 3:**
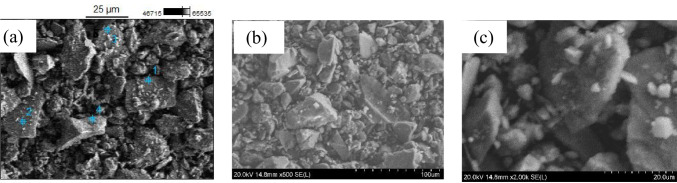
**a**–**c** SEM image for dust with various magnifications of the desert-dust particles collected from 0.5 × 0.5-m^2^ glass panels located horizontally at 10-m distance from the Earth surface, Urgench, Uzbekistan

Absence of systematics in the dust particle size and shape is common for dust particles collected in different geographic locations (Dunhuang; China; and Doha, Qatar, (Aïssa et al. 2016, Ilse et al. 2019, Javed et al. 2017)). SEM micrographs of dust particles (Fig. 3 (a)-(c)) confirm presence of various particle shapes, sizes, and morphologies. The small size particles are hardly seen. The largest particle size in the SEM image is ~ 10 µm. The shape of the particle is one of the important indicators showing the possibility of agglomeration of the dust particles. Nearly spherical shape particles are not visible in the SEM mage. Irregular shape of the dust particles that are abundant indicates that they cannot be attached to each other effectively as the inter-particle forces will be small enough. On the one hand, that reduces the possibility of agglomeration of the particles into heavier ones. On the other hand, the possibility of wind-induced dust transfer to long distances will be enhanced.

**Fig. 4:**
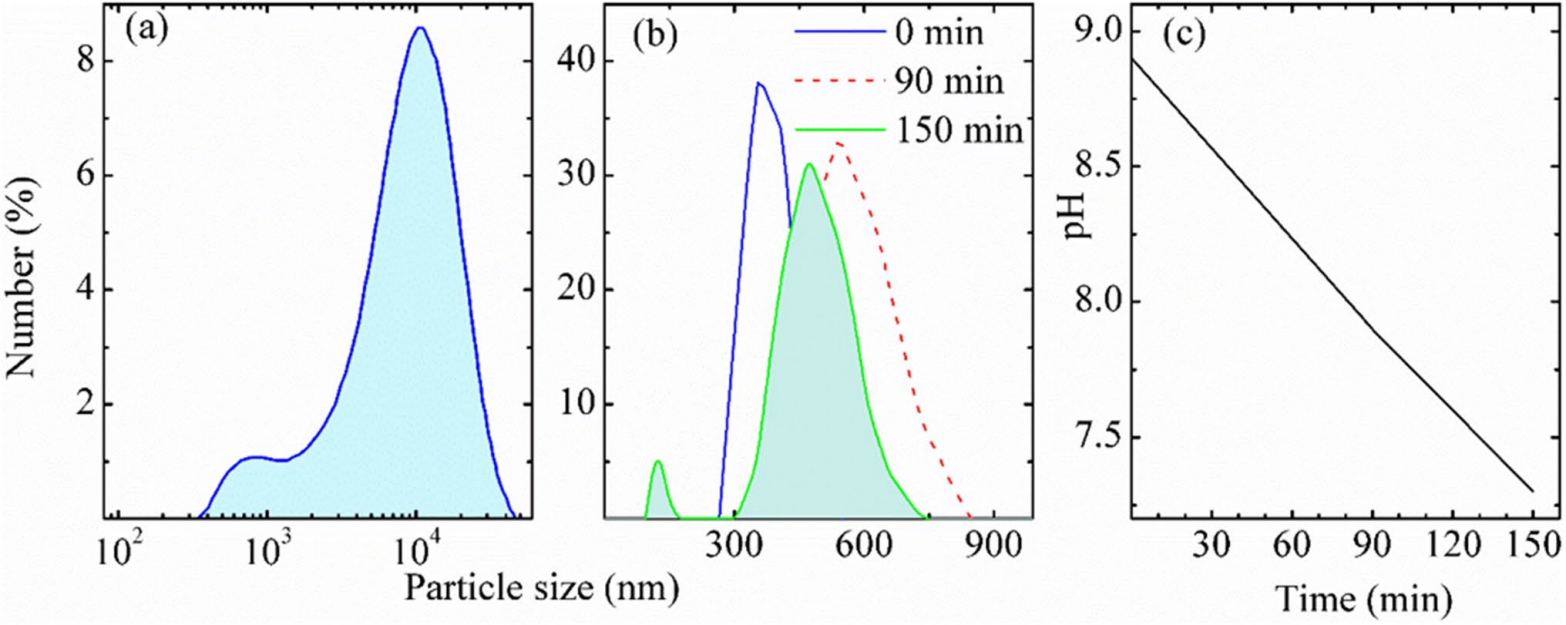
Particle size distribution of the dust in DI water measured by **a** Mastersize and **b** Zetasizer at different times: (―) right after mixing the dust with DI water, (―) 90 min later, and (―) 150 min later, and **c** kinetics of change of pH in DI water

The particle size in the SEM images (Fig. 3) is consistent with the results obtained by dynamic light scattering measurements using the instrument Mastersize (Fig. 4(a)) that showed particle size measurements with a maximum around ~ 10 µm. Smaller size particles have also been detected (Fig. 4(a)) with maximum around 600 nm, which is in excellent agreement with the results of Zetasizer instrument (Fig. 4(b)). The smallest particles dispersed with DI water are of size ~ 150 nm (Fig. 4(b)). Figure 4(b) shows the results obtained at different times: right after the dust was dispersed with DI and after 1.5 and 2.5 h after the dispersion. Analysis shows that size distribution of particles changes with time, because of, probably, chemical reactions of some of the salts in the dust with DI water. Such reaction might influence on pH of the dispersion. Figure 4(c) shows time dependence of pH of the dispersion of the dust with DI water. The result can be explained by reaction of CO_2_, CaO, MgO, Na_2_O, and P_2_O_5_ with DI water that changes acidity of the dispersion. Dust particle size in the range from 150 nm to 300 µm is another common feature of the dust collected at different geographic locations. Presence of different size and shape dust particles complicates maintenance of PV modules. If the PV module glass is not texturized, then the dust particles can be removed from the surface by cleaning. However, if the glass is texturized with the texture size of micrometer or nanometer scale, suppressing light reflection from glass might create comfortable conditions for the dust particles to stay there for longer time. Although the measurement shows particle size is of few hundreds of nanometers that can be of wire type particle that can enter the nanometer scale wall of the texturized glass. If that is the case, it will be difficult to remove such particles from the glass surface. Upon long time exposition of such texturized glass without cleaning or after dust storm, the nanosize voids of texture can fill out with smaller size dust particles that might drastically reduce the PV module efficiency.

## Dust composition, structural and vibrational properties

Composition of the soiling has been studied by XRF. First, by using the molecular weight of the oxides, weight of each element as a percentage for that molecule has been found. Then, by dividing the percentage of each oxide to molecular weight, the molecular proportions of each oxide have been found. By knowing the percentage of each element in one molecule, one can calculate the total amount contributed by the number of molecules in the XRF analysis and the amount of O contributed by various oxide molecules. From the proportions of theses weights of elements and of oxygen, one can calculate elemental percentage for the whole rock or mineral. The constituting compounds have been established and presented in Table 1 from highest to lowest atomic concentrations. Analysis shows that the dust particles have different concentrations of elements and compounds. Large amount of quartz (SiO_2_), CO_2_, CaO, Al_2_O_3_, Fe_2_O_3_, MgO, and Na_2_O, has been detected. Smaller number of other compounds are present also. Among the detected particles, CO_2_, CaO, MgO, Na_2_O, and P_2_O_5_ react with water and change acidity of the dispersion, which explains the reason of particle size change with time (Fig. 4(b)). The rest of the compounds such as SiO_2_, Al_2_O_3_, and Fe_2_O_3_ do not react with water at ambient conditions. Since there is no local nearby industry, influence of “local emission sources” into the dust composition is not substantial.

**Table 1:** Dust composition from XRF analysis. Background intensity (BG) is provided

Analyte	Result	Line	Net Int	BG Int	Ref. (Groundwater et al. 2012)	Ref. (Aïssa et al. 2016)
SiO_2_	43.1812%	Si-Ka	56.520	0.153	66.9	
CO_2_	27.2997%	C-Ka	0.263	0.048		7
CaO	9.6567%	Ca-Ka	56.679	0.138	3.7	
Al_2_O_3_	9.5312%	Al-Ka	16.288	0.534	21.9	
Fe_2_O_3_	4.5938%	Fe-Ka	33.074	0.111	4.2	
MgO	2.9889%	Mg-Ka	1.883	0.045	4.3	
Na_2_O	1.0634%	Na-Ka	0.290	0.006		
TiO_2_	0.7717%	Ti-Ka	0.879	0.015	2.2	
P_2_O_5_	0.3324%	P-Ka	0.724	0.034		
Cl	0.2052%	Cl-Ka	0.430	0.080		
SO_3_	0.1380%	S-Ka	0.272	0.025		
MnO	0.0775%	Mn-Ka	0.433	0.069		
ZrO_2_	0.0505%	Zr-Ka	1.969	0.799		
Cr_2_O_3_	0.0444%	Cr-Ka	0.153	0.036		
SrO	0.0410%	Sr-Ka	1.566	0.582		
ZnO	0.0245%	Zn-Ka	0.359	0.135		
CaCO_3_						58
Al_2_(SiO_4_)O						17
Mg_2_SiO_4_						9
Ca_2_MgSiO_7_						8

Quartz is the common element observed by all researchers that have studied dust particle composition. Dust compositions collected in many locations, such as Gandhinagar in Ahmedabad, India (Bergin et al. 2017) and in Arizona (Groundwater et al. 2012), are similar to our findings. However, proportion of molecules in and composition of dust particles depend on geographic location. Different elements have been observed in Qatar (Aïssa et al. 2016, Javed et al. 2017) such as Al_2_(SiO_4_)O, Mg_2_(SiO_4_), Ca(CO_3_), Al_2_(SiO_4_)O, Ca_2_Mg(Si_2_O_7_), and Mg_2_(SiO_4_).

**Fig. 5:**
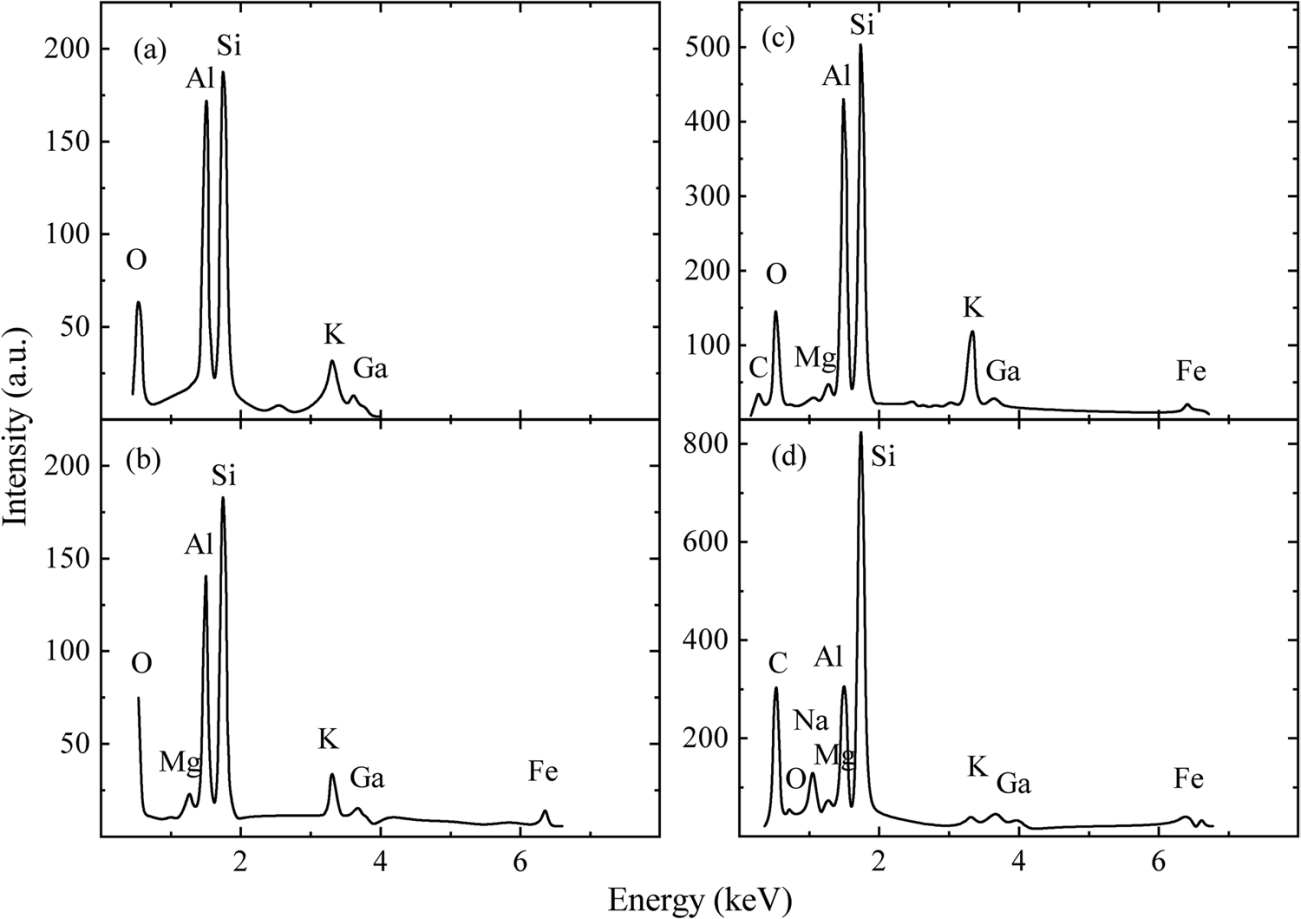
Compositional analysis by EDS for dust that fell on glass for four different spots (**a**)–(**d**) of the image in Fig. 3(a)

Elemental compositional analysis has been performed by EDS for the four different places of image in Fig. 3(a) and the constituting chemical elements have been established (Fig. 5). Large concentrations of elements such as C, O, Na, Mg, Al, Si, K, Ca, and Fe have been detected. The results are consistent with those of XRF results (Table 1) and of inductively coupled plasma atomic emission spectroscopy that detected substantial amount of Ca, Al, Fe, K, Mg, and Na. The other elements such as Ti, P, Mn, S, Ba, Sr, Zn, Zr, V, Co, Cr, and Cu are also present in smaller amount. The results are consistent with EDX and XRF measurements.

**Fig. 6:**
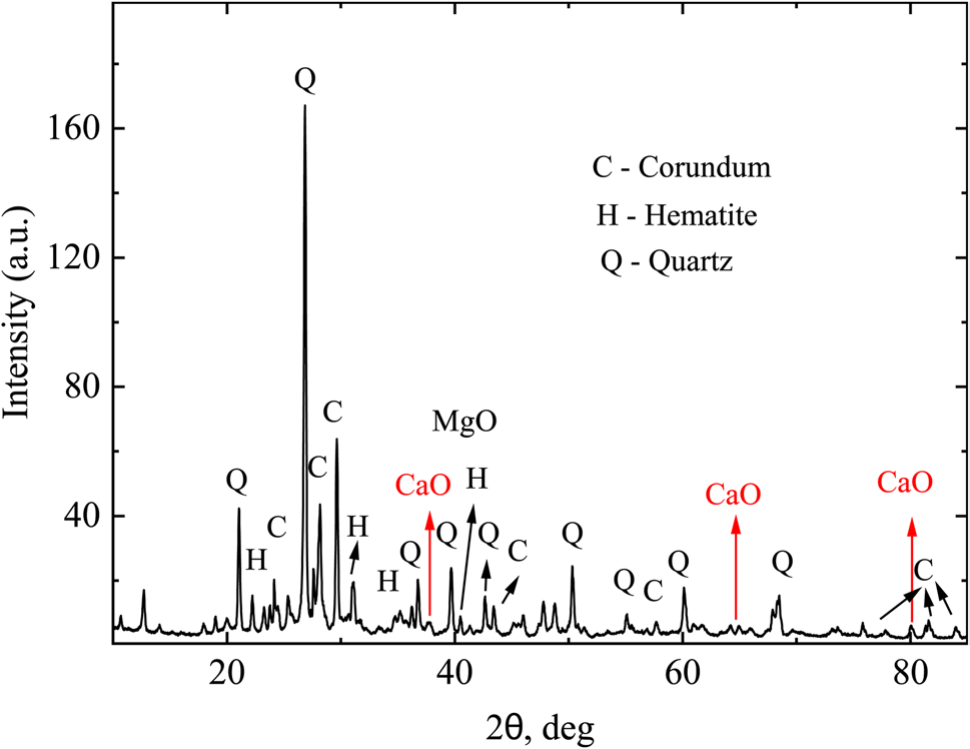
X-ray diffraction (XRD) spectra of the soiling collected from glass surface. Main peaks correspond to quartz (Wang et al. 2018), corundum, hematite (Lang et al. 2015), CaO, and MgO, the materials detected by compositional analysis

Figure 6 shows XRD spectra for soiling collected from the glass surface. One can see the peaks corresponding to quartz (Wang et al. 2018), corundum (Smrčok et al. 2001), hematite, CaO (Oftedal 1927), and MgO (Sasaki et al. 1979), the molecules detected in compositional analysis. Structural properties of the other materials observed in compositional analysis are not detected because either their concentration or particle size might be small and thus was not detected. Analysis shows that the peaks corresponding to quartz, hematite, and corundum are sharp, so these particles might be more crystalline although some level of disorder might be present.

**Fig. 7:**
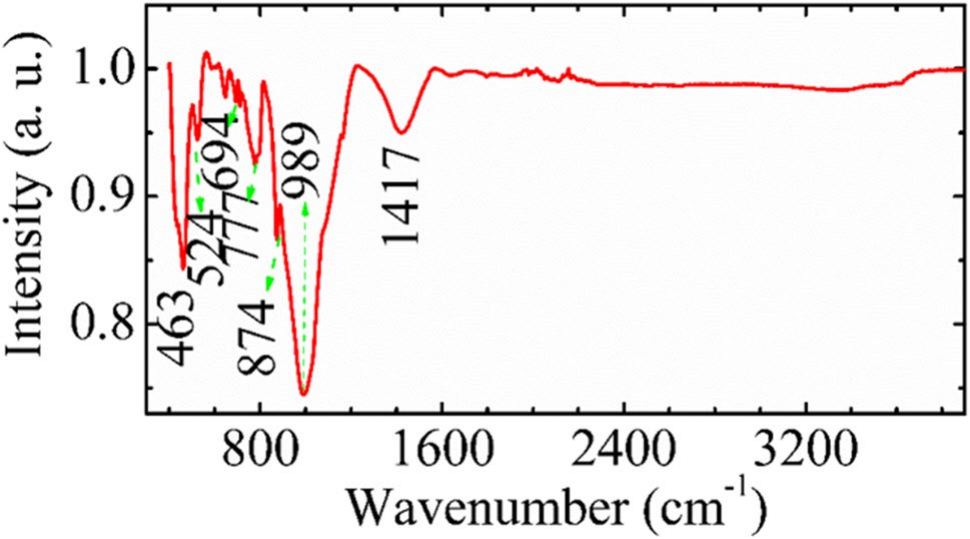
IR spectra of the dust collected from the glass surface. Main peaks corresponding to quartz and hematite are pointed out and analyzed

To study and identify chemical substances or functional groups in soiling, IR spectra have been measured in transmittance mode (Fig. 7) that gives the information related to the interaction between molecular bonds. According to elemental compositional analysis, quartz is the main component of the dust with the main characteristic absorption bands at 874, 777, 694, 524, and 462 cm^−1^ (Ojima 2003, Senthil Kumar and Rajkumar 2013). The Si–O bonds in the region 900 to 1100 cm^−1^ are the strongest bonds due to stretching whereas those in the range 400–800 cm^−1^ are due to bending. The absorption at 800 cm^−1^ is due to Si–O-Si symmetrical stretching vibration. The bands at 462 cm^−1^ and 524 cm^−1^ might be assigned to mixed Si–O-Si and O-Si–O bending modes. The peak at 694 cm^−1^ is close to that at 695 cm^−1^, which according to Ref. (Saikia 2014) belongs to Si–O symmetrical bending vibration. It arises due to the octahedral site symmetry and is the indicator of crystalline nature of quartz. The band at 777 cm^−1^ is due to the vibration in the tetrahedral site symmetry.

The peak at 524 cm^−1^ is the characteristic peak of hematite Fe_2_O_3_ (Yariv and Mendelovici 1979). It can arise due to the Fe–O stretching mode vibration. The intensity of the hematite peaks is strong, which is consistent with its large concentration observed from compositional analysis.

**Fig. 8:**
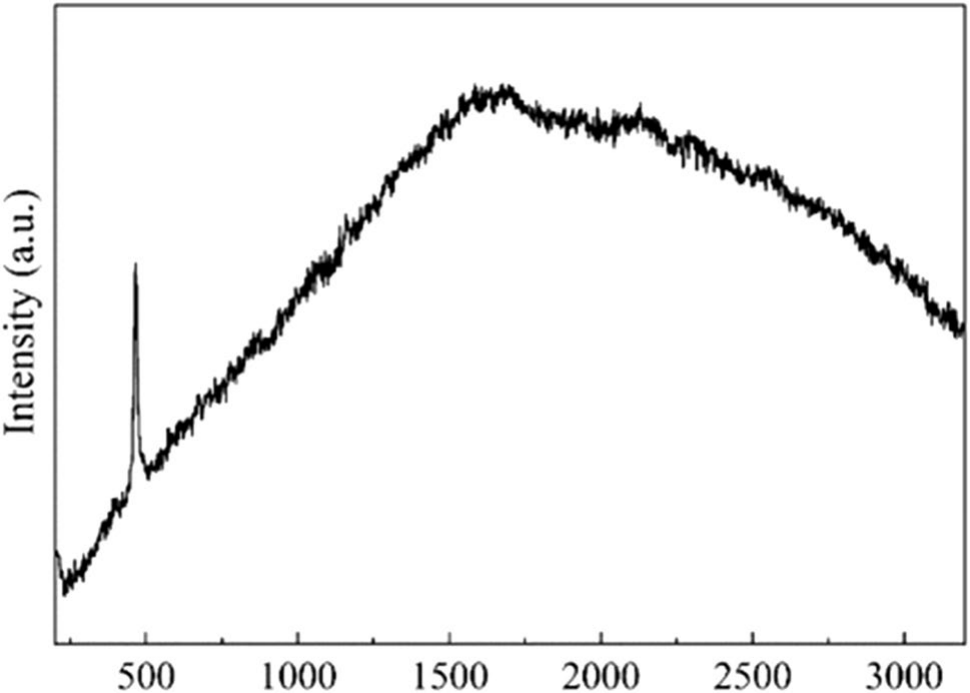
Raman spectra for the dust particles

Raman spectroscopy gives complementary information to the above discussed IR analysis. Figure 8 presents the Raman spectra for the dust. The Raman spectra analysis confirms the dust elemental composition based on Figs. 6 and 7 and supports well the compositional information presented in Table 1. The results are consistent with those of Ref. (Sobron et al. 2019) for quartz. One can see the peaks located at ~ 205 and 465 cm^−1^ that are characteristic to α-quartz (Sharma et al. 2011). This is expected as main part of the dust particles consists of quartz. The strong Raman line at 465 cm^−1^ is the fingerprint of Si–O-Si symmetric stretching modes of 6-membered rings of SiO_4_ tetrahedra in α-quartz. The peaks corresponding to hematite and corundum, etc. are not detected. However, the results must be taken with a certain care since this technique is rather qualitative because of the small diameter ∼1 µm of the probed zone whereas some particles are smaller size then the beam diameter. Furthermore, Raman peaks of other compounds can be too weak and/or embedded within the recorded Raman spectrum of the dominant species (i.e., quartz).

## Influence of dust dispersed in water on optical properties of glass and human being

**Fig. 9:**
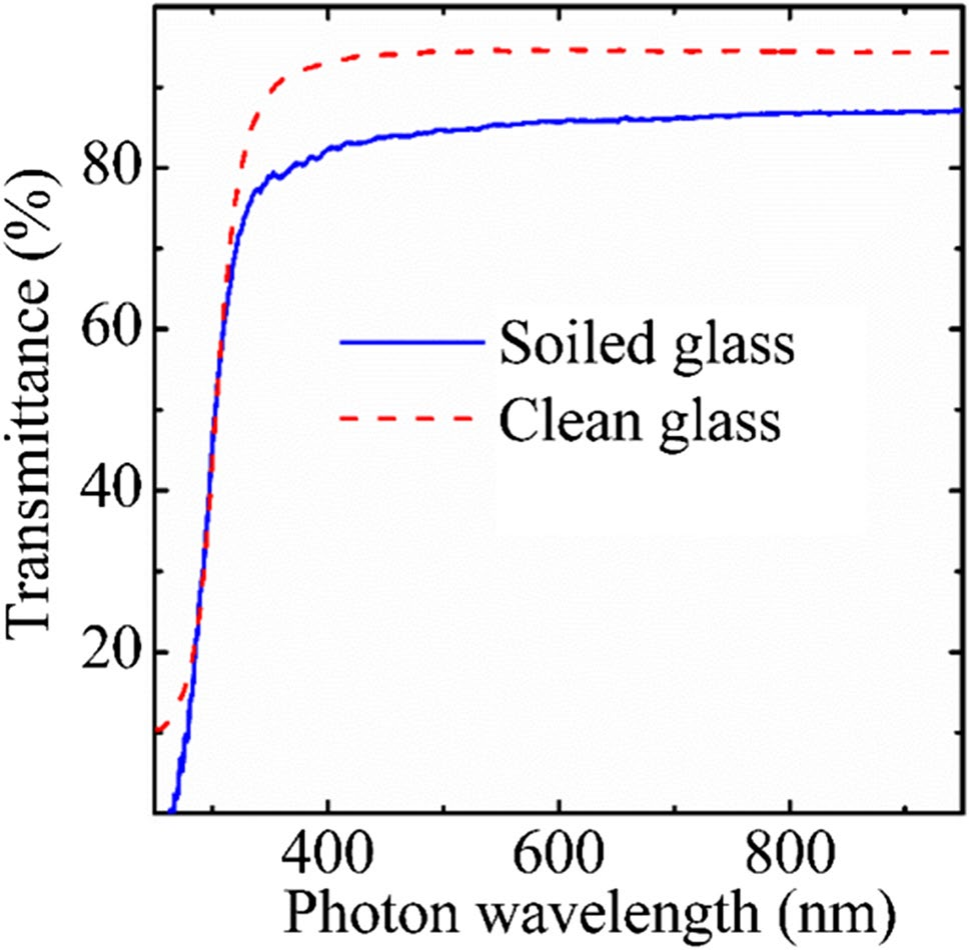
Transmittance spectra of wet glass with dust collected within 2 days with short raining on the 3rd day

Dust strongly influences optical transmittance of glass. Figure 9 presents transmittance spectra of the glass after dust fell on it within 2 days with subsequent raining that changed optical properties of glass. Although the transmittance is reduced to about 10%, it was within 2 days only. Flat glass commonly reflects ~ 8% sunlight (Liapis et al. 2017). If the dust reduces transmittance to 10%, in total ~ 23% of sunlight will not reach solar cells, which is critical for solar modules. To reduce sunlight reflection to < 2%, surface of glass for solar panels should textured. Based on the particle size measurements (Fig. 4 (a), (b)), one can say that the texture size cannot be on micrometer scale as substantial amount of the dust particle is submicrometer size. Enhanced deposition of those particles is expected to the micrometer scale textured glass surface. This indicates that different design of texture size of the solar panel glass and method of cleaning should be selected. The natural dust-induced reduction of the transmission of photons in UV-far infrared part of the photon energy is even, which is strongly related to physical and chemical properties of the dust.

These analyses indicate necessity of multifunctional anti-soiling coatings on the solar module glass that minimize the light reflection and repel the dust particles. Furthermore, material development for self-cleaning system that removes contaminants from a PV module surface by means of an automatic, water-saving, and labor-free process is an important challenge (Sun and Böhringer 2020). Knowledge of the particle composition might be useful in selection of materials for anti-soiling coatings for the glass encapsulating the PV module.

Analysis of the particle crystallinity and size can be useful to evaluate influence of the dust on human being. The inhalation of the disordered or crystalline silica can create the lung disease (Krivacsy and Hlavay 1993, Mohamed and Paleologos 2018). The concentration of this quartz is proportional to the toxicity. Due to the presence of quartz in dust, the inhalation of the quartz particles in the size of 0.5–0.7 µm may produce diseases such as chronic silicosis, acute silicosis, accelerated 15 silicosis, and silica tuberculosis (Keramydas et al. 2020, Ross et al. 1993). Presence of quartz in the air might be a carcinogenic agent (Downward et al. 2017, Keramydas et al. 2020). The inhalation of hematite along with other substances in the dust might create lung disease. Large concentration of hematite nanoparticles of size ∼14 nm in doses 50–400 mg/L is reported (Pariona et al. 2017, Tombuloglu et al. 2020) to be phytotoxic for barley.

## Conclusion

In summary, physical and chemical properties of dust in the Pre-Aral region of Uzbekistan such as Republic of Karakalpakstan and Khorezm have been studied systematically. The major and minor constituent substances present in the samples have been identified by X-ray fluorescence spectroscopy, energy dispersive X-ray diffraction, and inductively coupled plasma optical emission spectroscopy. Presence of the substances such as quartz, CO_2_, hematite, lime, corundum, and magnesia as the major constituents and several other trace materials has been established in the dust particles. Particle size measured by Mastersize and Zetasizer is in the range 20 nm–10 µm. From X-ray diffraction studies, the peaks corresponding to quartz, hematite, and corundum are found to be more crystalline with some level of disorder. Analysis of influence of the dust particles on human has been performed. Disordered or crystalline quartz can cause the lung disease; the particles in the size of 0.5–0.7 µm may produce diseases such as chronic silicosis, silicosis, and silica tuberculosis whereas hematite might create lung disease. Quartz and hematite are harmful to the human beings and are the deciding factors for pollution. Inhalation of the substances is known to lead to various respiratory diseases that will be huge in the case of pregnant ladies. These forms of disease are progressive with a continuing decrease of lung function even in the absence of further dust exposure. The result provides valuable information about quality of atmosphere that might be possible sources of the abovementioned and/or other diseases. We found that the dust particle size can be further reduced in water, which is accompanied by reduction of pH, which can be explained by reaction of CO_2_, CaO, MgO, Na_2_O, and P_2_O_5_ with water that changes acidity of the dispersion. Dust particles polluting the glass reduce its optical transmittance and thus are expected to worsen the solar panel performance. Particle size measurements performed provided useful information for selection of proper design of texture of solar panel glass for reducing light reflection and enhancing dust repelling.

## Data Availability

Upon request, the data will be made available to readers.
